# Teaching Emotional Intelligence: An Academic Course for Hospital Teachers

**DOI:** 10.5334/cie.13

**Published:** 2020-01-30

**Authors:** Meirav Hen

**Affiliations:** 1 Tel-Hai Academic College, IL

**Keywords:** Hospital teachers, Hospitalized children, Emotional Intelligence, Academic course

## Abstract

Hospital teachers work in a unique educational milieu that serves hospitalized children. In order to meet these children’s educational needs, teachers are expected to display high emotional abilities that will allow them to be creative, flexible and innovative, and able to work in distressing situations. For this reason a 30-hour Emotional Intelligence academic course for hospital teachers was developed and conducted, based on the revised theoretical framework of Mayer, Caruso & Salovey (2016). This mixed methods research study examined 50 hospital teachers who participated in this 10-week course, using a pre- and post-questionnaire, focus groups, semi-structured interviews and a final paper with a reflective summary. All training materials and examples were geared towards working with hospitalized children. Findings indicated an overall increase in the ability of hospital teachers to identify, understand and regulate their emotions from the beginning to the end of the training. Further results indicated that teachers felt the course increased their emotional self-awareness and understanding of emotions, as well as empathic feelings towards their students. In addition, they felt that the course was too short, and that they needed more practice in order to master these emotional abilities. This research contributes to the growing literature on the importance of Emotional Intelligence skills in teachers, and specifically in teachers who work in hospitals and other special education settings.

All school-aged children are entitled to obtain their education in a school setting (Jennings & Greenberg, 2009). This recommendation exists not only because of legal mandates, but also because of the social and developmental advantages the school setting provides children, including those with special needs (Bisogni et al., 2015; Ratnapalan, Rayar & Crawley, 2009). Some children, by virtue of acute or chronic medical conditions, are unable to attend school on a regular basis (Forrest, Bevans, Riley, Crespo & Louis, 2011). They are either at home or hospitalized and their educational needs are met in out-of-school circumstances (Capurso, 2014).

In order to support the development and educational needs of hospitalized children, hospital schools were established by law first in England and the United States (Wiley, 1987) and then in many pediatric and general hospitals around the globe (Goodman, 1988). In Israel, following the special education laws (Israel Ministry of Education, 1988), the law for the education of sick children was approved in 2001 (Vorgan, 2006). This follows the assumption that every child should have the best possible start in life through a high-quality education, which allows them to achieve their full potential (Israel Ministry of Education, 2001). Therefore, a child with health issues should have the same opportunities as his/her peers, including a broad and balanced curriculum (Gabbay, Cowie, Kerr & Purdy, 2000).

Teaching in hospital schools in Israel is carried out mostly by qualified teachers and is officially based on the curriculum of a regular school (Vorgan, 2006). Hospital teachers are required to deliver the curriculum as fully as possible in a hospital setting, often working at the child’s bedside, taking into account the physical and emotional state of the student, competing with the demands of health professionals, visitors, families and assorted distractions (Andreatta, Robol, Bolognanai & Dodman, 2015; Hopkins, 2016). Proponents of hospital schools believe that the academic work has an important role in the lives of hospitalized children, that the topics and methods as well as the presence of the teachers themselves play a very important therapeutic role which supports the medical treatment (Maor & Mitchem, 2018). The academic process offers the children a chance to continue their studies and obtain grades; it gives them faith in their own abilities, keeps up school readiness and satisfies cognitive and social needs, at least partially (Capurso & Dennis, 2017; Eaton, 2012).

Goodman (1988) argues that the teacher, a familiar figure for most hospitalized children, can become a mediator between the child and the medical staff through the establishment of a close personal relationship with the child. This relationship may help to increase the child’s overall cooperation with treatment, and to some degree free him from fear and pain (Goodman, 1988). Teachers must be psychologically oriented, use their ability to observe and be intuitive in order to employ the proper methods and words to motivate the hospitalized child (Hopkins, 2016). They are expected to be familiar with developments in the emotional, educational and medical fields relevant to their students’ progress and treatment (Csinady, 2015).

Although hospital teachers have been working officially in Israeli hospital schools for almost two decades, only recently have their professional needs been the focus of research (Hen, 2018). Hospital teachers in Israel, like in many other countries, do not receive training specific to this unusual setting (Benigno, Fante, Epifania, Caruso, & Ravicchio, 2018), and therefore constantly feel the uncertainty and unfulfilled professional needs in practice (Hen, 2018). Following this literature and the understanding that hospital teacher’s work in a unique educational setting that requires a set of emotional skills that will allow them to professionally meet the educational needs of the hospitalized child (Bisogni et al., 2015), a specific 30 hour emotional intelligence academic course for hospital teachers was developed and researched. The course was based on an experiential teaching model and included: 1) In-depth information about the four emotional intelligence skills as described by Mayer, Caruso & Salovey, (2016) 2) Knowledge of how emotional intelligence skills play an integral role in stress management, self-regulation, decision-making, interpersonal relationships, team building and the overall quality of one’s professional life (Zeidner, Matthews & Shemesh, 2016) and 3) Innovative strategies and tools to increase each emotional intelligence skill using activities, simulations and group discussions (Brackett, & Katulak, 2007). All training materials and examples were geared towards working with hospitalized children.

## Emotional Intelligence

Emotional Intelligence (EI) was proposed as a cognitive model to understand how rational thinking is enlisted to solve emotional and interpersonal problems among people (Salovey & Mayer, 1990). It was defined as a mental ability or trait that allows a person to process emotional information accurately and effectively, and thus to regulate and manage emotions intelligently (Mayer, et al., 2016). The important expression of EI is the balance between emotion and mind, and the ability to regulate the emotions, beliefs, and inner states of one-self and others in order to provide useful information for judgment and action (Zeidner, Roberts, & Matthews, 2008). EI was described as the ability or capacity to identify, perceive, express, understand, use, manage, and care for emotions so that they promote a person’s personal growth and sense of well-being (Zeidner, et al., 2016).

Many studies have examined the functional and adaptive value of EI, and have found a close connection to professional and academic success (Baczyńska & Thornton, 2017; Joseph, Jin, Newman & O’Boyle, 2015; Miao, Humphrey & Qian, 2017; Garg, Levin & Tremblay, 2016), to subjective well-being (Sánchez-Álvarez, Extremera & Fernández-Berrocal, 2016), physical and mental health (Fernández-Abascal & Martín-Díaz, 2015) and life satisfaction (Extremera & Ray, 2016). People with high EI were found to be optimistic, warm and empathic, and to possess a consistently high level of self-esteem (Song et al., 2010). Furthermore, a significant positive correlation was found between the level of EI and self-efficacy among teachers, physicians, social workers, and businesspeople (Harper, 2016; Hen & Goroshit, 2011).

Goleman (1998) proposed a theory of performance that builds on the basic EI model as a means to predict personal effectiveness at work and in leadership. He suggested a set of guidelines that could help people in the workplace develop the skills required for working effectively with others. Empowering EI can give a person a higher sense of self-efficacy and contribute to one’s functioning (Warwick, Nettlebeck & Ward, 2010). Researchers who studied EI following long-term and short-term training programs argued that the best way to examine the increase and its effects is through combined quantitative and qualitative research, as the only way to incorporate and learn more about the complexity of this ability (Corcoran & Tormey, 2012).

## EI Training

The literature indicates that high levels of EI among teachers contributes to their professional functioning, protects them from burnout, reinforces job satisfaction, and improves their relationship with students (Brackett, Palomera, Mojsa-Kaja, Reyes, & Salovey, 2010; Wong, Wong & Peng, 2010; Zysberg, Orenshtein, Gimmon & Robinson, 2017). Studies show that teachers and pre-service teachers are eager for EI studies and show increased EI scores following long-term and short-term courses (Dolev & Leshem, 2017; Hen & Waler, 2012; Vesely, Saklofske & Nordstokke, 2014). In some studies, increased EI was associated with increased well-being, emotional self-efficacy and educational competence (Hen & Sharabi-Nov, 2014; Vesely, Saklofske & Leschied, 2013).

The study programs described in the literature range from focused courses (10–12 academic hours) to several day-workshops that include an in-depth study of the EI model, and how these abilities are expressed in academic learning, problem solving, decision making, classroom management, interpersonal relationships, and team building (Brackett et al., 2010; Hen & Waler, 2012; Vesley et al., 2013, 2014). Courses also focus on how participants can increase their emotional skills through group activity, understanding relationships, simulations, and discussion (Dolev & Leshem, 2017). Teachers, education students, and principals who participated in these workshops and courses reported greater self-awareness of their own emotions and the emotions of others, and improved feelings about themselves and their relationships with colleagues, students, and parents (Corcoran & Tormey, 2012; Dolev & Leshem, 2017; Kasler, Hen & Sharabi-Nov, 2013).

In a model developed by Nelson, Low & Nelson (2005) based on Transformative Learning, the authors emphasized five stages of learning that employ self-observation, increased self-awareness, self-understanding, and the understanding of self-development and self-improvement. These stages are taught through experience, reflection, and individual and group tasks. Their results showed short-term improvement in achievement of those students whose teachers had undergone the workshops (Nelson, Low & Nelson, 2005). Another study, which examined the contribution of an EI movement course to pre-service teachers, found that EI increased at the end of the course, and their students reported more meaningful learning and teaching experiences during the course than at its beginning (Hen & Walter, 2012). No EI courses were found in the literature for teachers working in special education or with special-needs populations, such as sick and hospitalized children.

## The Present Study

Based on the literature review highlighting the unique work of hospital teachers and their specific need for high emotional skills, this study examined the contribution of an academic EI course to the perceived emotional skills and professional functioning of hospital teachers.

The main research question was: In what way, and to what extent, would a 30 hour academic course in emotional intelligence for hospital teachers contribute to the increase of the perceived emotional intelligence skills of hospital teachers at work?

It was hypothesized that this academic course in EI would increase hospital teachers’ perceived emotional skills such as: Appraisal and expression of emotion, Regulation of emotion and Utilization of emotion and would contribute to their perceived emotional ability and professional satisfaction at work.

## Method

### Participants

The study involved 50 teachers (48 women and 2 men) from hospital schools from all parts of the country. Of these, 32 teachers held a bachelor’s degree in special education and a teaching certificate, 18 had a master’s degree in special education/educational counseling or sciences. The age of participants ranged from 28 to 62 years, with a mean age of 37 (SD = 5). The number of years of seniority in the teaching profession ranged from 2 to 32 years, with a mean of 12 years (SD = 6). The course was offered as one in a battery of academic courses offered to teachers. Hospital teachers elected to enroll in the course and received academic credit from the Ministry of Education for their participation in the course, as they do in all courses offered by the Ministry.

### Design

This was a mixed methods study that utilized an EI questionnaire, focus groups, interviews and course summary papers. It was designed to study the contribution of an EI academic course for hospital teachers from the beginning to the end of the course, and three months afterwards. The course was based on theory, experience and reflection as recommended in the literature (Corcoran & Tormey, 2012; Hen & Goroshit, 2011; Hen & Sharabi-Nov, 2014), and focused on relevant contents to teaching in hospitals. The course took place in a mid-sized educational college in central Israel and included three academic hours per week for ten weeks (30 academic hours). The course was led by a clinical psychologist who is an EI expert, and a former hospital teacher. During the learning processes hospital teachers were encouraged to share some emotional experiences from their everyday work and analyze them in class with supervision and peer support.

### Procedure

In order to capture a wide picture regarding the nature and development of emotional skills in hospital teachers, this study utilized both quantitative and qualitative research methods. The participants in the present study gave their informed consent, and were then asked to fill an EI questionnaire (Schutte et al., 1998) at the beginning and the end of the course. In addition, data was collected from three focus groups at the beginning, midway and at the end of the course; twenty-eight semi structured interviews with the participants during the course; and participants’ final papers.

### Instruments

**Emotional Intelligence Questionnaire** (Schutte et al., 1998) This is a self-report questionnaire based on the Salovey and Mayer model (1990), and translated into Hebrew (Hen & Goroshit, 2011). The questionnaire consists of 33 statements, 13 of which examine the ability to evaluate and express emotions (e.g. “emotions are one of the things that are the spice of life”), 10 items examine the ability to regulate and control feelings (e.g. “I control my feelings”), 10 items examine the ability to benefit from emotions, such as using emotions to solve problems (e.g. “When my mood changes I can see new opportunities”). The participant is requested to specify to what extent each statement describes him/her on the Likert scale, with 1 indicating “definitely disagree” and 5 indicating “definitely agree”. The reliability of this test as measured by Schutte et al. (1998) was: α = 0.90, test-retest reliability at two weeks’ interval was 0.78. In the present study the entire questionnaire was utilized and a high internal consistency reliability was found for the total number of items, α = 0.83. The reliability of the first factor “the ability to assess and express feelings” was α = 0.86. The reliability of the second factor was “ability to regulate and control emotions” was α = 0.73 and the reliability of the third factor “the ability to benefit from emotions” was α = 0.77.

**Three focus groups** at the beginning, midway and end of the course. This research method was chosen in addition to interviews in order to allow more data to accumulate from the group processes and interactions (Kitzinger, 1995). It was assumed that because participants came from different hospital schools around the country, the group interactions would provide us with unique information. Focus groups involved two stages. At the first stage all participants were divided into four small groups and were requested to discuss a specific matter for one hour. At the second stage members from the small groups shared their experience and conclusions with the large group. Conclusions from all groups were discussed by all participants in the large group and main conclusions served as the data for analyses for each focus group. The first focus group centered on describing their work as teachers in hospitals and the perceived emotional skills required of them at work. The group also discussed hospital teachers’ specific needs and their expectations of the course. The second focus group midway through the course discussed the emotional skills learned so far in the course and how they could be applied in their work as hospital teachers. The third focus group at the end of the course was an attempt to examine whether the emotional skills learned in the course provided a sufficient and appropriate response to the teachers’ needs for their work with hospitalized children.

**Semi structured Interviews** – During the course, three students of the Master’s degree program in Educational Psychology from an academic college in Northern Israel conducted interviews with 28 participants who had agreed to be interviewed. The purpose of the personal interviews was to learn about the personal emotional development of the participants in the course, their learning experience and how well they are able to apply the skills learned during their course of work. The advantage of this tool (interview) is its ability to move from structured questions that ensure uniformity on the one hand, to open questions that give a personal character to the conversation, and to provide the interviewee with freedom of expression that helps us understand his/her personal perceptions (Shkedi, 2003). As is customary in this kind of study, the interview questions guide was only an initial starting point, and during the interviews, additional questions were asked to deepen the understanding of the phenomenon described (Hill et al., 2005).

**Course final papers** – submitted about three months after the course and constituting a final document for the course itself and its contribution to teachers’ work in the field. In this summary paper, the teachers were required to take a case from the field and analyze it with the help of the theory and skills learned. In addition, they were asked to reflect and record their own thoughts about the course, along with a summary.

### Data processing method

The quantitative data were analyzed using a t-test for dependent samples. The qualitative data were analyzed using the CQR method, by three coders separately, with the aim of adopting a number of perspectives on the aggregation of the central themes arising from the data (Hill et al., 2005). The coders were five students who completed a bachelor’s degree in psychology and education. Each coder analyzed all the qualitative data and defined domains and core ideas. In the second stage, the three coders met and conducted a cross-analysis in which they tried to characterize domains and core ideas that can describe the interviews, while trying to reach a consensus. The domains and core ideas formulated during this phase were transferred to the chief researcher who served as an auditor and she went over the data in order to identify the biases created in the group thinking process. Next, all the ideas that were formulated were grouped into categories and themes, and their frequency was counted.

According to Hill and colleagues (2005), if a category or a theme appeared in more than half of the participants (in our case over 25 participants), we termed it ‘typical’ and identified it as **most participants** who responded in a certain manner. Themes cited by fewer than half the participants, but more than 10 participants, were termed as ‘some’ and identified as **some participants** who responded in a certain manner. Low-frequency topics (up to 10 participants) were associated with similar themes (Hill et al., 2005), and sometimes, if important information was added, the findings were presented in the results section. In the final stage, all the qualitative materials were re-examined by the author in order to determine their analysis in an exhaustive manner.

## Results

Based on the hypothesis that the levels of EI would be high at the completion of the course compared to its beginning, a dependent-samples t-test was conducted (see Table 1).

**Table 1:** Mean standard deviations and paired-samples t-test of Emotional Intelligence questionnaire by time of measurement.

	Beginning of the course (n = 50)		End of the course (n = 50)		t_(49)_	p	Cohen’s d

	M	SD	M	SD			

Emotional Intelligence	3.61	0.39	3.70	0.35	2.22	0.031	0.243
**Subscales**							
Appraisal and expression of emotion	3.49	0.36	3.59	0.25	2.18	0.034	0.323
Regulation of emotion	3.79	0.50	3.88	0.49	2.01	0.050	0.182
Utilization of emotion	3.54	0.51	3.64	0.47	2.03	0.048	0.204

A dependent-samples t-test was conducted to compare overall EI and its components between the beginning and completion of an EI academic course for hospital teachers. Table 1 shows there was a significant difference in the scores for overall EI from the beginning (M = 3.6, SD = 0.39) to the end of the course (M = 3.7, SD = 0.35); (t(49) = 2.223 p = 0.03).

In addition a difference in all subscales of EI was found between the beginning and the end of the course: Appraisal and expression of emotion (M = 3.5, SD = 0.36) vs. (M = 3.6, SD = 0.25); (t(49) = 2.181 p = 0.034), Regulation of emotion (M = 3.8, SD = 0.50) vs. (M = 3.88, SD = 0.49); (t(49) = 2.010 p = 0.050) and Utilization of emotion (M = 3.54, SD = 0.51) vs. (M = 3.67, SD = 0.47); (t(49) = 2.031 p = 0.048).

These results suggest that overall perceived EI as well as all its components can be increased in hospital teachers who participate in a 10week academic EI course.

In order to further examine the nature of hospital teachers’ need for emotional skills, and the contribution of an academic course in EI tailored to their presented needs, a qualitative set of data was collected and analyzed.

**Qualitative results:** An analysis of the qualitative data revealed twelve themes grouped into five main categories. The categories and themes will be presented as **Category 1:** describing the work of the hospital teachers in the educational centers of the hospitals (themes: teaching context, teaching demands, teaching context, job satisfaction); **Category 2:** expectations from course participation (themes: learning about myself, learning about the feelings of the other); **Category 3:** the learning experience in the course (themes: learning about emotions, becoming aware: emotions that arose during the course); **Category 4:** the applied aspect of learning (themes: application as it relates to me, application with respect to the other); **Category 5:** the learning outcomes of the course (themes: from theory to practice, practice in the perspective of time).

Hill et al.’s (2005) criteria for reporting the occurrence of the responses was used in reporting the data (See data processing section, p. 11). In addition, some basic demographic information was included when teachers were quoted in the following section. The term “young teacher” refers to teachers who have been working in the hospital less than 3 years. “Experienced teachers” are those who have been working in the hospital more than 10 years.

### Categories and Themes

#### Category 1: Nature and quality of educational work with hospitalized children

*Teaching context*: Most of the teachers (above 25) described their work with hospitalized children as very different experience from teaching in a regular school in the community. Hospitalized students are admitted to the hospital to receive medical treatment and therefore the entire environment is geared towards that goal. Medical and therapeutic procedures take center stage in the hospital and have a clear priority over educational activity. Learning is not demanded, it is basically the child’s and his parents’ choice. Hospital teachers in our study do not have a regular work plan or a fixed timetable; instead, their schedule is determined on a daily basis according to the needs of the children who are hospitalized at that day. Some teachers meet the child once or twice because he/she has been hospitalized only for a very short time, and others, especially in departments of chronic, oncological, or psychiatric illnesses, see the children for longer periods of time. In both cases, the schedule of hospital teachers is very unstable and erratic. The following quotes were chosen from the first focus group.

“The educational work in the hospital is very difficult to describe when you are not there … it is not at all like teaching in the regular school in the community … There is no agenda, no program … we improvise all the time.” (Young teacher)

“Children come from different places, different situations, and with different needs… and not to mention their parents… and we need to gently and professionally find out what their situation is and to adjust their educational activities.” (Experienced teacher)

*Teaching demands*: The teachers maintained that they needed to be proactive, creative, flexible, and able to make immediate contact, make quick decisions, and adapt themselves to a large number of hospitalized children of different ages, cultures and medical conditions. In addition, they need to respond to the children’s parents, the medical staff and the overall situation.

“The work is complicated because you have to take into consideration so many parties…. the children, their parents, doctors and nurses, and sometimes community teachers…” (Teacher, Interview)

“You need to be focused at all times…you can’t just talk foolishly or say things you are not expert in. You have to be very precise and ethical …” (Teacher, Interview)

*Teaching contents*: Teachers reported their work is divided between regular classroom content (i.e. English and mathematics), lessons specific to hospitalization (e.g. preparation for medical procedures), creative and expressive lessons for stress reduction and pain processing (e.g. drawing, music, puppet theater), and lessons in social skills (i.e. values, traditions, cultural holidays, and social events).

“Sometimes we simply teach a regular lesson, sometimes we work in creative and expressive materials and sometimes we prepare a child for a medical procedure … It’s hard to know what the day will bring… we arrive at the department and get organized…”(Experienced teacher, Interview)

*Job satisfaction*: In general, most of the teachers described their work positively as challenging, interesting, satisfying, inspiring, contributing and meaningful. But they also described it as stressful, very intuitive, painful, intensive, overwhelming and difficult to manage. The quotes are from the first focus group:

“I feel like it’s a wonderful job, but complex and multifaceted …. Happiness and suffering mix all the time …” (Experienced teacher)

“The most accurate word for me is that this is a really challenging job – it’s very, very interesting and difficult … I come home exhausted.” (Young teacher)

“You see a child who has just been diagnosed with cancer … he doesn’t really understand what’s going on, but his parents are so scared, and you want so much to comfort them and say it’s going to be okay.” (Teacher)

#### Category 2: Expectations from course participation

*Learning about myself*: The teachers expected the course to answer two main emotional difficulties in their work – one related to themselves: preserving the energy level to keep up with the fast and changing pace of the work (burnout), learning to contain and process the great pain they face, improving the ability to make professional and not only intuitive decisions, to improve the ability to recognize, understand, and express their own emotions (awareness and insights) and to conduct themselves emotionally in a professional manner.

“It’s really strange that most of our work is working with a special population and under special conditions and we do not get a framework that will help us to manage emotionally with full awareness and professionality.” (Young teacher, Interview)

*Learning about the feelings of others*: the ability to better understand the emotional needs of hospitalized children and their parents, and strategies for addressing these needs (empathy), to understand complex emotional situations, especially with parents and medical staff, to provide professional solutions with educational tools in emotional situations with a hospitalized child, to give children and parents tools for emotional relief.

“I need emotional tools that will help me understand myself, the children and their parents and other people around me.” (Teacher, Interview)

#### Category 3: The learning experience in the course

*Learning in the course*: Most of the teachers spoke about the course being the first professional forum where they could talk directly about their emotions and where they could experience emotional situations and tools. They talked about the fact that in the hospital both the doctors and the paramedical therapists are always supervised on their internal processes, while they are not used to receiving any emotional guidance, but only instruction in teaching materials. Most of them described the study of emotions as extremely interesting, and the experience as refreshing but also not easy. Experiential learning greatly helped to process and better understands the emotions that arose in the course, also the fact that the facilitator raised many examples. They declared that it did not feel like a regular theoretical course at university, but more like training with theoretical aspects. All quotes were chosen from the second focus group:

“The theoretical material was very interesting to me but I really did not feel like being at university again … I wanted tools.” (Teacher)

“I felt that I lacked the more dynamic guidance … I’m always jealous of the therapists and doctors who are constantly working on their inner world.” (Teacher)

“… In the course I got a sense of in-depth learning; we constantly explored ourselves and our learning and did not remain only at the level of conceptualization.” (Experienced teacher)

*Emotions that arose during the course*: At first the participants described feelings of shame and fear in facing themselves and others, describing embarrassment and wanting to glamorize the things they said about themselves, they felt judgmental towards themselves and others, but said that the empathic and nonjudgmental atmosphere of the course helped them open up and feel more confident. The specific work on the identification of negative emotions, especially feelings of fear and helplessness, was recorded by the majority of participants as the most significant learning, as well as learning about decision making. All quotes were chosen from the second and third focus group:

“It seems to me that I finally learned the difference between awareness and observation: awareness is the *knowledge* I have about myself, while the observation is the *understanding* that I have of myself … I usually got stuck in the awareness because observation forces you to understand and change.” (Teacher)

“In the group and personal experiences I felt naked…I had to see myself in a mirror and look deeply within…it was scary!” (Young teacher)

“I suddenly realized that when I feel afraid, I tend to repress or distance myself from it and not understand it, and I need to learn to deal with it so that it doesn’t hurt me or others at work or at home. …” (Experienced teacher)

#### Category 4: The applied aspect of the course

*The application with regard to myself*: Most of the teachers described that, especially at the beginning of the course, they felt so loaded down with concepts and theory that they came to the field feeling confused – as if the course damaged their work rather than helped. Suddenly they became very conscious and preoccupied with observation, and felt a difficulty in containing all of this and being available to the children. They felt that what they used to do in a very intuitive way was now being done in a conscious manner and not spontaneously. Towards the middle of the course, the teachers began to report that they were already able to identify and understand many of their own feelings that they had not really noticed before, did not identify and certainly did not know how to name. They reported that perceiving and conceptualizing feelings forced them to think about where these feelings came from – for example: “What threatens me?” or “What annoys me?” – then deal with both the emotion and the situation. They reported that understanding an emotion is much more complex than identifying it and requested repeated training on the subject of understanding emotion. They explained that using emotion to focus thought or to increase motivation was a new conscious strategy for them and it was much easier for them to mobilize thinking in order to regulate emotion within (proportioning). Nevertheless, in order to understand what others feel, they were able to mobilize emotion and felt more empathic, especially towards parents. All quotes were chosen from the third focus group:

“During the course I found myself wiping tears, excited, restless, and especially feeling a lot of excitement.” (Teacher)

“As we progressed, I felt a great deal of difficulty dealing with concepts related to my inner world; I felt that I was losing my connection to the purpose of the course and I could no longer distinguish between those things.” (Teacher)

“I experienced emotional upheaval. Sometimes I felt completely disconnected from the group discourse. I felt that I did not understand what they were talking about, and sometimes I felt that the information was inside me, and that the connection suited my inner world and that I was just being reminded of things.” (Experienced teacher)

“The course brought about a flood of feelings, memories and different doubts in my life. I went through thought processes from the point of view of the teacher/therapist and sometimes from the perspective of the student/patient.” (Experienced teacher)

*The application regarding others*: The part that most of the teachers felt easier to implement understood the emotional feelings and needs of the children. They reported better understanding the difference between fear and anxiety in the children, identifying helplessness and pain, even when a child is belligerent or withdrawn. They were able to see pain processing in children, help the children describe pain and feelings of excitement or embarrassment, and to think together with the child of ways in which to express and cope. Many of the teachers felt that they asked the children fewer questions and yet responded in a way that encourages the child to talk and share. Still, in the middle of the course most of the teachers claimed that the emotional communication with the parents felt ‘stuck’ and needed to be processed.

“I must note that I had mixed feelings during the course. At first I was full of curiosity, and slowly I also felt tension – how would I incorporate this knowledge as a teacher? Will I know how to regulate myself emotionally? How will I cope? The pressure did not just go away, but with time and the accumulation of knowledge, I feel that there is a process of development. I am learning to accept criticism and not to be hurt, to try to process my experiences in a practical and reflective manner.”(Young teacher, Third focus group)

#### Category 5: Learning outcomes in the course

*From theory to practice*: Toward the end of the course, the interviews and the focus group showed increased awareness and recognition of the significant process they experienced in the course, along with the ability to identify the emotions that arise within them. In addition, they reported a better understanding of the effectiveness of the emotional tools they received during the course, and the initial ability to better understand the educational interaction in the hospital from their point of view and from the perspective of the child and the parent. There was no uniformity in the sense of what they received during the course, but most of the teachers felt that they gained knowledge to which they had never been exposed previously.

Some of the teachers reported the many emotional experiences, focus groups, small-group work, and dilemmas that were raised, helped them turn theory into practice, but still felt that the course was only an introduction. They claimed to have learned emotional strategies, such as interpreting a child’s pain or fear, or not being intimidated by their own helplessness or that of others, and not to run around asking a lot of questions or giving advice. They learned to recognize their own pressure and that of others, and place it on a temporary ‘hold’, rather than to immediately act on it (i.e. ‘to be’ rather than ‘to do’). At the end of the course, most of the teachers maintained they had acquired emotional skills and strategies, but still needed a lot of learning in order for these to become professional emotional abilities.

“I think that the course contributed to my ability to breathe and relax in difficult coping situations. Even if I do not have an immediate solution, I know that it is best to take a moment, stop, think, consult, and process emotions and then formulate a plan of action or response.” (Teacher, Interview)

“I think I am learning to be more forgiving with myself and to be able to generate positive criticism from mistakes. Moreover, I feel that I am able to maintain self-criticism processes within myself, not only after the act.” (Teacher, Third focus group)

“When we came to talk about the concept of containment, I was happy. My understanding of this concept was really important to my continuation as an educator. I never really understood what it is to “contain”, today with the help of the course I understand that to include the other side is to allow myself to feel what the other side feels, to let the other’s emotions be within me, and then to activate the processing of my emotions.” (Young teacher, Third focus group)

*Practice in the perspective of time*: The majority of participants noted that writing the final paper helped them to better integrate the theoretical material they learned in a perspective of time. They wrote in reflection that they felt much more aware of emotional processes within themselves and others, but the application still required a lot of attention and preparation, and even then it did not always feel accurate enough. Some of the teachers felt the course contributed to their professional functioning very significantly by providing conceptualization and a theoretical framework to their intuitive work. They noted they felt more professional and more well-defined to themselves and to other health professionals and clients. Some of the teachers said they intended to look for a continuation course, and others claimed they had returned to or started emotional therapy or some type of private emotional guidance. All the participants claimed the course opened a window to their emotional world, and it is no doubt impossible to work as a teacher in the hospital without high emotional skills and abilities. The final papers showed that most of the teachers were able to understand the theoretical part of the course and knew how to use the theoretical concepts learned, but demonstrated a limited practical ability. All quotes are from final papers:

“For me the course was a link between personal awareness processes and my professional consolidation as an educator. I am happy about the processes I am going through, on both the personal and professional levels. I have no doubt that every process of development and growth that I am undergoing will improve not only the quality of my personal life, but also my abilities as an educator.” (Teacher)

“I also feel that the knowledge gives me the tools to understand and deal with educational challenges that used to be frustrating for me, and have now become a positive challenge (more emotionally than rationally) because I know that I can cope and understand, and I am not powerless. The knowledge and the tools have been absorbed into my blood and since I believe in their power, I have faith and a sense of my capability.” (Experienced teacher)

## Discussion

Teachers in hospitals work in a unique educational environment that requires a high level of emotional abilities (Andretta et al., 2015). They work with children hospitalized for short and long periods of time and are required to be proactive, creative and flexible in order to provide an educational solution adapted to the needs of these children (Äärelä et al., 2016, 2018; Hopkins 2016). Their teaching is often more directed at alleviating anxiety, encouraging coping with hospitalization and increasing cooperation with the medical staff. It is often less focused on the transfer of knowledge or the development of learning skills, and thus requires much more emotional skill (Eaton, 2012; Wiles, 1987). On the other hand they do not receive any extra emotional training, assuming they will develop these skills along the way (Csinády, 2015).

The academic course on EI presently studied was developed for teachers in hospitals, based on need, and generated from other academic courses in EI found in the literature (Dolev & Leshem, 2016; Hen & Sharabi-Nov, 2014). As recommended in the literature, the present study utilized quantitative and qualitative research methods (Corcoran & Tormey, 2012) to examine the increase in the level of perceived EI from the beginning to the end of the course, and the contribution of this course to the well-being and overall job satisfaction of 50 hospital teachers who participated in the course.

Overall findings in the present study suggested that hospital teachers, like teachers in other studies, express a need for “emotional tools” in order to better conduct their educational work (Dolev & Leshem, 2016) with a focus on the unique teaching demands they encounter as hospital teachers (Andretta et al., 2015). Similar to other studies, teachers in the present study self-reported a significant increase in emotional abilities from the beginning to the end of the course (Hen & Sharabi-Nov, 2014). However, in the qualitative findings a gap between the “learning of emotions” experiences and the ability to practice emotional skills in vivo was identified. As proposed in other studies, teachers in the present study maintained that learning emotional skills is a long-term process that starts with knowledge and awareness, but requires time and support in order to internalize and practice these skills at work (Hen & Goroshit, 2011). Hospital teachers in the present study stressed how difficult it is to identify and deal with feelings of fear and anger, and to generate thought in order to regulate these feelings as suggested in the literature (Meyers et al., 2016).

Further qualitative findings demonstrated how much the nature of the educational work with hospitalized children requires good emotional skills, and how little supervision hospital teachers receive on this subject. The literature often touches this issue, but rarely addresses what can be done in order to improve this situation (Hen, 2018). Our findings also suggested that the learning process of emotional skills is challenging for teachers, the “emotional language” is often unfamiliar, and the need to explore their inner world is difficult. This of course supports the notion presented in the literature that in order to actually develop teachers’ emotional skills, a long-term and experiential teaching method is recommended (Brackett & Katulak, 2007; Nelson, Low & Nelson, 2005). Although the studied course was relatively long-term and practice was engaged in the learning process, at the end of the course hospital teachers felt the course was just a “good start”.

Thus, a heightened awareness of the emotional process, conceptualization, and a longing to deepen their understanding occurred when the course began with the theoretical discourse on emotions and EI in general and later when the content connected to the teachers’ emotional world (Hen & Walter, 2012). All of these apparently caused teachers to report a significant change in EI, even if the qualitative findings showed that only a partial development of emotional abilities was reported. This, of course, highlights the importance of integrated research and the ability to better understand the significance of quantitative self-reporting (Corcoran & Tormey, 2012).

The qualitative data collected in this study supported the quantitative findings showing an increased level of perceived self-awareness to feelings and processes of emotion recognition and expression. It also contributed to the ability to identify better the feelings of hospitalized students and their parents. Findings suggested the course raised teachers’ awareness and need to understand their feelings and those of others, but also raised the level of confusion and difficulty that arises from learning strategies to understand emotion (Jennings & Greenberg, 2009). Throughout the course and at its conclusion, teachers reported that they still needed a lot of training in order to truly understand the emotions they experience at work, what aroused them, and how to deal with them. As with other courses mentioned in the literature, the teachers in this course reported that the material was important, interesting, and that experiential learning and reflection assisted learning to a great extent (Brackett & Katulak, 2007; Corcoran & Tormey, 2012). They also reported that learning combined with field practice contributed to the ability to make the transfer from theory to practice (Dolev & Leshem, 2017).

## Conclusion

Undoubtedly, the current study reinforced the view that academic courses on the management and regulation of emotions should be introduced into the training programs for teachers and preservice teachers, especially those working in special education frameworks. Also reinforced by the findings of this study was the fact that an academic course alone, not accompanied by fieldwork and significant processing, contributes to theoretical learning and understanding of EI, but does not seem to allow for meaningful implementation. This appearss to become even more critical in complex work environments such as hospitals, where teachers are required to have high emotional skills and abilities to successfully cope with the lack of routine, teaching during illness, and the pain of meeting sick children and their parents (Eaton, 2012). It also appears the duration of learning in the course (ten weeks) was significant and made it possible to experience with the knowledge and not only engage in academic learning. It is still important to note that teachers completed the course with the feeling if there were no continuation or further systematic guidance in the emotional field, the awareness and strategies they learned would not develop into a tailored practice.

## Limits of the Study

Alongside the importance and contribution of these findings to the field of emotional discourse in education and teaching in general, and among teachers in the hospital in particular, the findings should be interpreted while considering several limitations. Firstly, the sample in this study was relatively small and perhaps not representative, since it was an elective course and only hospital teachers who wanted the course registered for it. Perhaps they do not represent the hospital teachers who did not register and do not feel the need for a course on emotions. In addition, there was no comparison control group in this study and all the quantitative and qualitative tools used to collect the data were self-reporting tools, which may at times be influenced by biases or social desirability. In addition, this study did not examine the characteristic or demographic aspects of the participants (such as gender, age, working and training experience, school ward), which might have shed more light on the findings. It also did not specifically examine the various aspects of the course and its practical contributions to teachers and students over time, but rather referred to its contributions during and immediately after the course. Finally, our study focused only on teachers’ experience during the course and shortly after, with no further on-going data collection on their self-growth and implementation of knowledge in their everyday practice. Furthermore, no data on teachers’ development or practice was collected from other sources such as hospitalized children, their parents or medical staff.

## Implications for Further Research

Despite the limitations of this research it is important to note that the study examines an important process of increasing the emotional discourse in the field of education in general, and among teachers in special education in particular. The research literature recognizes the need to increase and sustain emotional discourse both as a way of improving the quality of life and functioning of teachers and to increase these abilities among students – but few studies examine practical ways to do so (Brackett & Katulak, 2007, Nelson et al., 2005). Many teachers are not familiar with the emotional discourse, have never been trained in it, and in the face of the complex educational reality they feel helpless and frustrated, sometimes finding it difficult to provide an appropriate emotional response to their students (Jennings & Greenberg, 2009). This becomes even more significant and crucial when teachers work in an educational space with unique characteristics in which their students are hospitalized children. These children are in an uncommon emotional situation and need an appropriate response to their educational needs, but also to a process that promotes recovery (Csinady, 2015). It is hoped that further research in this field will help to develop academic courses in EI and other emotional skills for teachers in general and especially for teachers in special education. Further research in the specific and unique educational milieu of hospital teachers should more specifically explore their exact emotional needs on taking into consideration different personal and demographic characteristics; and evaluate different methods of increasing the emotional discourse and the implementation of emotional knowledge in the work place as perceived by peers, colleagues and clients.

## Appendix 1

**Table 2:** Syllabus: EI training for hospital teachers (By Dr. Meirav Hen and Mrs. Maskit Gilan Shochat).

Class no.	Hours	Subject	Teaching Method

1	3	**Introduction: The science of Emotional Intelligence.** *Readings*: Mayer, J. D., Caruso, D. R. & Salovey, P. (2016). The Ability Model of Emotional Intelligence: Principles and Updates. *Emotion Review, 8*(4), 290–300.	Lecture & Discussion
2	3	**The four-branch ability model of Emotional Intelligence.**	Lecture & Discussion
3	3	**Emotional Intelligence and Education.** Reading: Keidar, D. (2015). Emotional intelligence and education. *Studia Edukacyjne*, 37, 327–348.	Lecture & Discussion
4	3	**Ability Model: How to identify, understand and regulate emotions in oneself** Readings: Hen, M. & Goroshit, M. (2011). Emotional competencies in the education of mental health professionals. *Social Work Education, 30*(7), 811–829. https://doi.org/10.1080/02615479.2010.515680	Experiential (Play roll, reflective, focus groups, film)
5	3	**Ability Model: How to identify, understand and regulate emotions in others** Readings: Hen, M. & Sharabi-Nov, A. (2014). Teaching the teachers: emotional intelligence training for teachers. *Teaching Education*, 25(4), 375–390. https://doi.org/10.1080/10476210.2014.908838	Experiential (Play roll, reflective, focus groups, film)
6	3	**Emotional Intelligence and Decision making** Readings: Hess, J. D. & Bacigalupo, A. C. (2011). Enhancing decisions and decision-making processes through the application of emotional intelligence skills. *Management Decision*, 49(5) 710–721.	Experiential (Play roll, reflective, focus groups, film)
7	3	**Managing emotions in a unique workplace** Maurice J. Elias, Linda Bruene-Butler, Lisa Blum & Thomas Schuyler (2000). Voices From the Field: Identifying and Overcoming Roadblocks to Carrying Out Programs in Social and Emotional Learning/Emotional Intelligence, *Journal of Educational and Psychological Consultation*, 11:2, 253–272.	Experiential (Play roll, reflective, focus groups, film)
8	3	**Managing emotions: Working with parents** Oren, D. (2011). Parenthood Treatment. *Psychology*, 15–27. (In Hebrew).	Experiential (Play roll, reflective, focus groups, film)
9	3	**Managing emotions: Working with hospitalized children.** Readings: Hen, M., Aasllan, R., Ben-Yizhak, S., Rish, M. (2019). Art therapy for hospitalized children. Academic Journal of Creative Arts Therapies, 9(1), 845–855 (in Hebrew).	Experiential (Play roll, reflective, focus groups, film)
10	3	**The art of practicing Emotional Intelligence–From theory to Practice** Readings: Brackett, M., & Katulak, N. (2007). Emotional Intelligence in the Classroom: Skill-Based Training for Teachers and Students. In: Improving emotional intelligence: A practitioner’s guide (pp. 1–27).	Experiential (Play roll, reflective, focus groups, film)
